# Supplementary material for: Genome-Wide Characterization and Phylogenetic Analysis of GSK Genes in Maize and Elucidation of Their General Role in Interaction with BZR1
Source: Int J Mol Sci. 2022 Jul 22;23(15):8056. doi: 10.3390/ijms23158056 (PMC9330802; doi:10.3390/ijms23158056)
Supplement: Supplementary file 1 [file ijms-23-08056-s001.zip › ijms-1753950-supplementary.pdf]

Supplementary

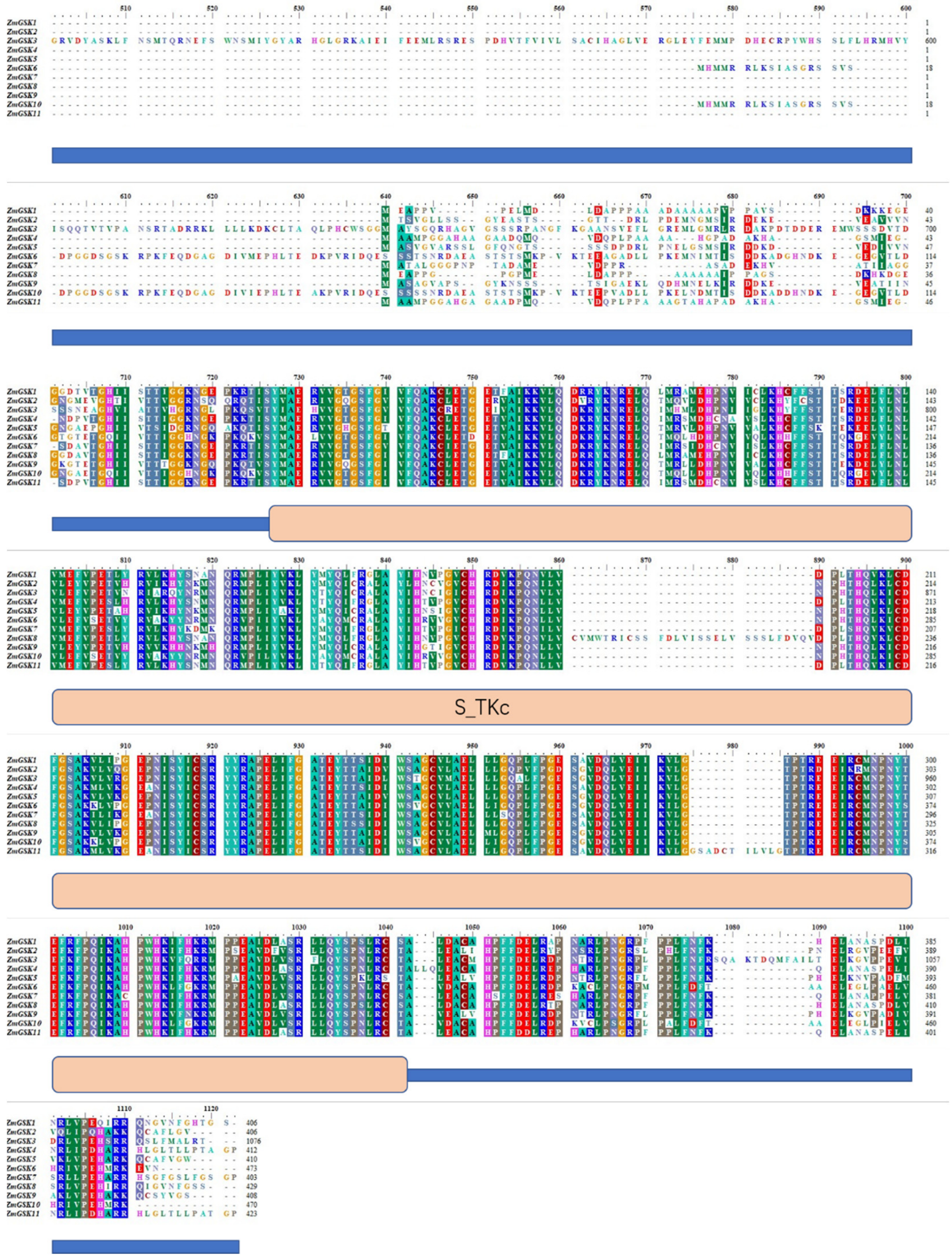

Figure S1 Sequence alignment analysis of maize GSK genes.

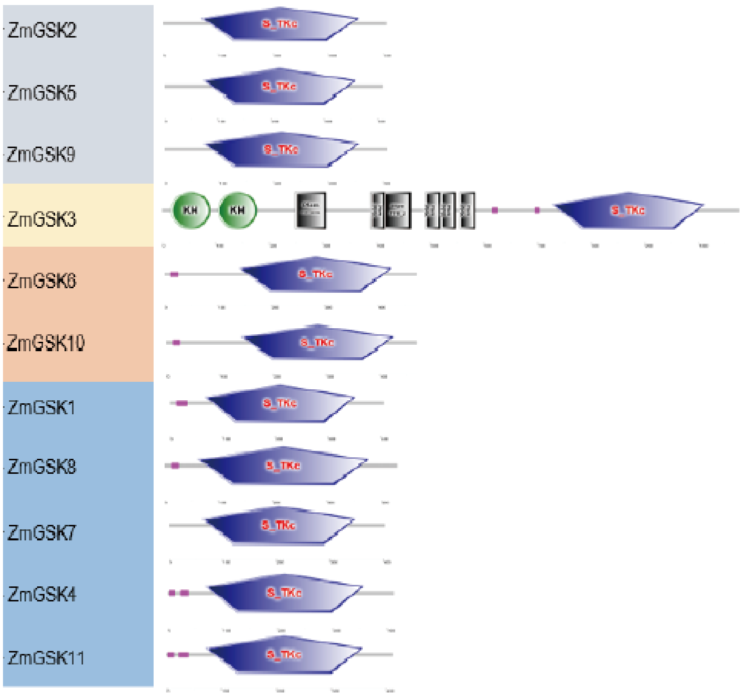

Figure S2 Conserved motif analysis of maize GSK genes.

Table S1. Conserved motifs in different maize GSK genes

Motif	Sequence
motif_1	TPTREEIRCMNPNYTEFKFPQIKAHPWHKIFHKRMPPEAVDLVSRLLQYS
motif_2	THQLKICDFGSAKVLVKGEPNISYICSRYYRAPELIFGATEYTTAIDIWS
motif_3	QTISYMAERVVGTGSFGIVFQAKCLETGETVAIKKVLQDKRYKNRELQTM
motif_4	KHYNNMNQRMPLIYVKLYMYQICRALAYIHNVPGVCHRDIKPQNLLVBPH
motif_5	RLLDHPNVVSLKHCFFSTTSRDELYLNLVLEFVPETVYRVL
motif_6	ALEACAHPFFDELRDPNARLPNGRPLPPLFNFK
motif_7	GCVLAELLLGQPLFPGESGVDQLVEIIKV
motif_8	VEGVVIEGNGAETGHIISTTIGGKNGZPK
motif_9	HELANAPPELVNRLVPEHARR
motif_10	PNLRCT
motif_11	MHMMRRLKSIASGRSSVSDPGGDSGSKRPKFEQDGAGDIVIEPHLTEDKP
motif_12	SYKPVKSTSAGADRLPDEMNEMSIRDDKE
motif_13	AEAPPAPDPMZLDQPLPAAAA
motif_14	VRIDQESSSSSNRDAEASTST
motif_15	MAAMPG

Table S2. Primers of GSK semi-quantitiative RT-PCR and qRT-PCR

Primer	Sequence
GSK1QF:	TCAACAACGAGCAGGGACG
GSK1QR:	GGTTCACCAGGAATCAGGACTT
GSK2QF:	ATGTGGCAGAGCGTATCGTG

GSK2QR:	CGCAGTTGTGAAGGTAAGCC
GSK3QF:	CTACAATGGCAATCTACAGGAGG
GSK3QR:	TGGTCTGGACTCTCTCTGCTG
GSK4QF:	GGTGTTTGCCACAGGGATG
GSK4QR:	TCCACAGCACTTTCGCCA
GSK5QF:	TTGAAACTGGTGAGACCGTAGC
GSK5QR:	TGCCAAGGCTCTACAAATCTG
GSK6QF:	CCTGTGCGGATAGACCAAGA
GSK6QR:	TGTTCCAGTCCCATCAAGTGTA
GSK7QF:	TTCCCGTCTTCTTCAGTATTCG
GSK7QR:	CCGAATCCTGAATGCCGT
GSK8QF:	ATCGTCTTCCAGGCTAAGTGTC
GSK8QR:	TCTGGTTCGCATTGCTGTAGT
GSK9QF:	AGTGAGCAGCCAGAGAACAGG
GSK9QR:	TTATGTGCCCAGTTTCGGTC
GSK10QF:	CTCTCGCTCTCCTCGGCTAA
GSK10QR:	CTGCTCCATCCTGCTCAAAT
GSK11QF:	TGGAGACTGGTGAGACTGTTGC
GSK11QR:	GAGTGGCATCCTCTGGTTCA
ACTINQF:	TCACTACGACTGCCGAGCGAG
ACTINQR:	GAGCCACCACTGAGGACAACATTAC

Table S3. Primers of clone GSK and BZR1

Primer Sequence	
GSK1F:	CAAGCAGCCAAGCAGTGTGAAC
GSK1R:	CAATATCACCGTCACCTGCAATC
GSK2F:	ACCCGTCGATTGTAGTTGCTCAG
GSK2R:	ATGCTACTGTGCGGTTAAACAGAAC
GSK3F:	ATGGAAGGCGTACCGCTCG
GSK3R:	CTAGGTCCTTAGCGCCATGAACA
GSK4F:	CGCCAAGAGAGAACAGGACACTAC
GSK4R:	GGTCAGATTCTACACCTTGAATTGC
GSK5F:	CCTTGGACAGCAGTTTGTGCTG
GSK5R:	TCATCGAGTCGTCATCCTCTTATCC
GSK6F:	TCTGAGGGAGGAGGATGCATATG
GSK6R:	CCGTCCACCTAATTCACTTCCTTC
GSK7F:	CACCGAGGAGAGAGAAGCCTGTC
GSK7R:	GCTACGGTCCACTCCCAAATAAG
GSK8F:	AGCGGCGTCTGAACTCTGAACC
GSK8R:	CTCCTAGCTGCTCCCGAAGTTGAC
GSK9F:	GAGGAGGTTACAGAGATGGCTTCAG
GSK9R:	AAGGCACGGTCATCTCACGATC
GSK10F:	CTGTGAGGGAGAAGGATGCATATG
GSK10R:	ACCGGTTCACTTCCTCATGTGTTC
GSK11F:	ACGGGAGAGAGGACGTTCATTTC

GSK11R:	AGCCTTCGCCTATGGTCCAGTAG
BZR1F:	GCTGCGTTCAGACGACCACT
BZR1F:	AGAGGAGCACCGTCCGAATA

Table S4. Primers of GSKs ligate to pGBKT7 and BZR1 to pGADT7

Primer Sequence	
GSK1BKF:	TCAGAGGAGGACCTGCATATGATGGAGGCGCCGCCG
GSK1BKR:	CCGCTGCAGGTCGACGGATCCCTAGCTCCCGGTATG
GSK2BKF:	TCAGAGGAGGACCTGCATATGATGACATCAGTAGGT
GSK2BKR:	CCGCTGCAGGTCGACGGATCCTCAAACTCCTAAGAA
GSK3BKF:	TCAGAGGAGGACCTGCATATGATGGAAGGCGTACCG
GSK3BKR:	CCGCTGCAGGTCGACGGATCCCTAGGTCCTTAGCGC
GSK4BKF:	TCAGAGGAGGACCTGCATATGATGGCAGCCATGCCG
GSK4BKR:	CCGCTGCAGGTCGACGGATCCCTATGGTCCAGCGGT
GSK5BKF:	TCAGAGGAGGACCTGCATATGATGGCCTCGGTGGGC
GSK5BKR:	CCGCTGCAGGTCGACGGATCCTCACCACCCTACGAA
GSK6BKF:	TCAGAGGAGGACCTGCATATGATGCATATGATGCGG
GSK6BKR:	CCGCTGCAGGTCGACGGATCCCTAATTCACTTCCTT
GSK7BKF:	TCAGAGGAGGACCTGCATATGATGGCCACCGCGCTC
GSK7BKR:	CCGCTGCAGGTCGACGGATCCCTACGGTCCACTCCC
GSK8BKF	TCAGAGGAGGACCTGCATATGATGGAGGCGCCGCCG
GSK8BKR:	CCGCTGCAGGTCGACGGATCCCTAGCTGCTCCCGAA
GSK9BKF:	TCAGAGGAGGACCTGCATATGATGGCTTCAGCTGGT
GSK9BKR:	CCGCTGCAGGTCGACGGATCCTCACGATCCAACATA
GSK10BKF:	TCAGAGGAGGACCTGCATATGATGCATATGATGCGG
GSK10BKR	CCGCTGCAGGTCGACGGATCCTCACTTCCTCATGTG
GSK11BKF:	TCAGAGGAGGACCTGCATATGATGGCCGCCATGCCG
GSK11BKR:	CCGCTGCAGGTCGACGGATCCCTATGGTCCAGTAGC
BZR1ADF:	GTACCAGATTACGCTCATATGATGACGAGCGGCGCC
BZR1ADR:	CAGCTCGAGCTCGATGGATCCTCAGGAAGGATCTGC

Table S5. Primers of GSKs ligate to p2913 and BZR1 to p3086

Primer Sequence	
2913GSK1F:	TCGAGCTCAAGCTTCGAATTCATGGAGGCGCCGCCG
2913GSK1R:	GCTCACCATCAGGATCCCGGGCTAGCTCCCGGTATG
2913GSK3F:	TCGAGCTCAAGCTTCGAATTCATGGAAGGCGTACCG
2913GSK3R:	GCTCACCATCAGGATCCCGGGCTAGGTCCTTAGCGC
2913GSK4F:	TCGAGCTCAAGCTTCGAATTCATGGCAGCCATGCCG
2913GSK4R:	GCTCACCATCAGGATCCCGGGCTATGGTCCAGCGGT
2913GSK7F:	TCGAGCTCAAGCTTCGAATTCATGGCCACCGCGCTC
2913GSK7R:	GCTCACCATCAGGATCCCGGGCTACGGTCCACTCCC
2913GSK8F:	TCGAGCTCAAGCTTCGAATTCATGGAGGCGCCGCCG
2913GSK8R:	GCTCACCATCAGGATCCCGGGCTAGCTGCTCCCGAA

2913GSK11F:	TCGAGCTCAAGCTTCGAATTCATGGCCGCCATGCCG
2913GSK11R:	GCTCACCATCAGGATCCCGGGCTATGGTCCAGTAGC
3086BZR1F:	TCGAGCTCAAGCTTCGAATTCATGACGAGCGGCGCC
3086BZR1R:	CACGCTGCCCAGGATCCCGGGTCAGGAAGGATCTGC
